# Effects of field enhanced charge transfer on the luminescence properties of Si/SiO_2_ superlattices

**DOI:** 10.1038/s41598-022-05566-4

**Published:** 2022-02-16

**Authors:** Deniz Yazicioglu, Sebastian Gutsch, Margit Zacharias

**Affiliations:** Laboratory for Nanotechnology, Institute of Micro Systems Technology – IMTEK, University of Freiburg, Georges-Koehler-Allee 103, 79110 Freiburg, Germany

**Keywords:** Nanophotonics and plasmonics, Quantum dots

## Abstract

The effect of an externally applied electric field on exciton splitting and carrier transport was studied on 3.5 nm Si nanocrystals embedded in SiO_2_ superlattices with barrier oxide thicknesses varied between 2 and 4 nm. Through a series of photoluminescence measurements performed at both room temperature and with liquid N_2_ cooling, it was shown that the application of an electric field resulted in a reduction of luminescence intensity due to exciton splitting and charging of nanocrystals within the superlattices. This effect was found to be enhanced when surface defects at the Si/SiO_2_ interface were not passivated by H_2_ treatment and severely reduced for inter layer barrier oxide thicknesses above 3 nm. The findings point to the surface defects assisting in carrier transport, lowering the energy required for exciton splitting. Said enhancement was found to be diminished at low temperatures due to the freezing-in of phonons. We propose potential device design parameters for photon detection and tandem solar cell applications utilizing the quantum confinement effect based on the findings of the present study.

## Introduction

Room temperature visible spectrum luminescence in quantum confined Si was reported as early as 1990^1^. Since then, Si nano crystals (SiNC) have been used as sensors and light emitting devices^2–5^. In such applications, the exploitation of the quantum confinement effect has allowed for tailoring the emission wavelengths and shifting the absorption edge^6–8^. One of the advantages of the SiNCs produced using the superlattice approach is the ability to directly grow NCs on the substrate along with an oxide layer providing a passive barrier against the spontaneous oxidation of Si^9,10^. In the case of solution synthesized NCs, this constitutes a technical limitation that would have to be overcome through the introduction of additional processing steps like passivation coatings and the construction of core–shell structures^11–13^. However, this advantage comes at a trade-off, wherein carrier transport through the superlattice is hindered by the dielectric^14^. This poses a hurdle to be overcome for applications such as current generation and electroluminescence where being able to run a current through the stack is imperative.

While SiNC based photovoltaic cells alone would not improve the industrial scale power generation efficiency of such devices enough to warrant the utilization of a more complicated production method, they can be used in tandem devices with bulk Si. In such applications being able to tailor the band gap using the quantum confinement effect could increase efficiency by addressing the non-absorption losses^15^. Such tandem devices have been realized, albeit with the top cell limiting the short-circuit current^16^. There remains a need for further studies of the exciton generation and charge transport mechanics at the NC oxide interfaces. Additionally, the ability to control the absorption band edge is promising for infrared sensing applications^17^. This could allow for all-Si monolithically integrated wavelength selective photo detector arrays. Such color imaging devices relying on the quantum confinement effect have been realized with Si nanowires but, to the best of our knowledge not with quantum dots^18^.

In order for a photo-current across the superlattice to be detected, excitons need to be split before recombination and the carriers need to be transported to the contacts^2^. Previously, the radiative lifetimes for SiNCs of similar size were experimentally determined to be 50 μs at RT and 250 μs at 80 K^19^. The transport of carriers through the dielectric in such systems has been studied experimentally^14^ and within the context of polaron transport theory^20^. In vacuum, electrons can move freely as long as there is no potential barrier they cannot overcome. However, the key difference in the case of transport through a dielectric where electron–phonon coupling is higher, is that an electron moving through the dielectric would polarize the medium, carrying its own polarization as it travels^21^. Furthermore, the charging of a NC by an electron would also produce a change in its energy from the neutral state^22^. This necessitates the energy level that an electron is migrating to be lower than the energy level of the state it had initially occupied. Such conditions can be achieved through the application of an artificial external electric field across the medium.

To this end we have studied the migration of photo-generated carriers through the superlattice under the effect of an external electric field of up to 2.5 MV/cm and as a function of the barrier oxide layer thickness (varied from 2 to 4 nm) with fixed NC size (3.5 nm). This was achieved by recording the emission spectra of the respective ensembles of SiNCs, analyzing the charge transport mechanics by investigating the change in photoluminescence (PL) intensity and comparing luminescence characteristics to electrical measurements.

## Methods

Two sample sets (passivated and non-passivated) comprised of twenty-five 3.5 nm SiNC layers with inter-layer-oxide barriers in between were prepared using a superlattice deposition process. The bilayer stacks were produced by alternatively depositing layers of silicon-rich-oxide (SiO_0.93_) and stoichiometric SiO_2_ through plasma enhanced chemical vapor deposition (PECVD) on Si substrates. The barrier oxide thicknesses were systematically varied from 2 to 4 nm by repeating the PECVD steps whereas the NC layer thicknesses were kept constant (3.5 nm). Additional thicker blocking oxide layers of 10 nm were also deposited at the top and bottom of the stacks. For current density measurements, samples with thinner (1 nm) inter-layer-oxide barriers and superlattices composed of fewer layers (18 bilayers) were fabricated. These samples were made without the 10 nm blocking oxide layers at either end to allow for carrier injection at lower fields.

The superlattices were annealed at 1150 °C for 60 min in inert atmosphere (N_2_) to phase separate the silicon rich oxide and the NCs. For the passivated sample set, an additional step in H_2_/N_2_ atmosphere was added for 60 min at 450 °C. This step was shown to radically reduce the density of the surface defects at the Si/SiO_2_ interface^23^. For transparent top contacts, 300 nm thick ZnO layers were added by atomic layer deposition. Aluminum layers of 300 nm were used for the other contacts, directly evaporated on top of the superlattices or on the back side of the highly As-doped wafers with resistivities measuring less than 5 × 10^−5^ Ω m.

The samples were mounted on a vacuum cryostat and PL spectra were recorded under the excitation of a 325 nm CW He-Cd laser with a power density of 0.65 mW/cm^2^. The measurements were carried out both at room temperature (RT) and at 80 K using liquid N_2_ coolant. To produce the field across the samples, a forward DC bias (accumulation regime) was applied at the contacts and the voltage was varied. Behavior in the inversion regime was not investigated since a rectifying behavior due to the lack of minority carriers was previously reported in similar structures^24^. It is significant that the field values reported are estimated average field values, calculated by modelling the NC layers as bulk media with average effective permittivity values. PL measurements with DC bias were done under zero current conditions. Current density measurements were done by applying a voltage ramp on a device analyzer and recording the current density, with and without excitation by a broadband light source.

## Results and discussion

**Figure 1:**
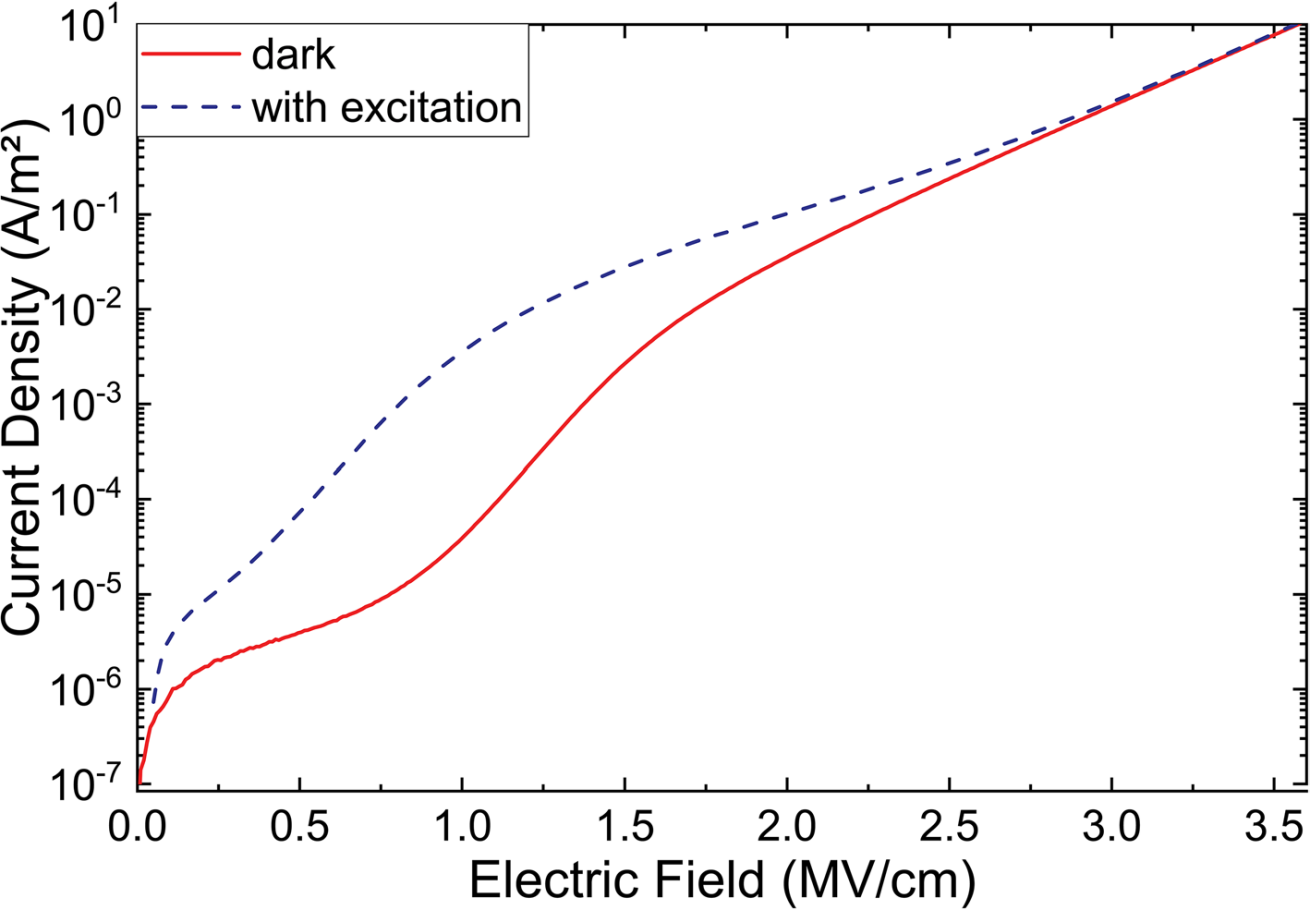
Field–current density plots for a NC superlattice of 18 bilayers, prepared without additional blocking oxide layers.

The current density plots in Fig. 1 show three different current regimes. There is an initial increase up to 0.1 MV/cm, where the sample under excitation reaches a higher value. This trend is followed by a slower rate of increase in current density for both cases, with the sample under excitation consistently measuring several orders of magnitude higher. Past 2.0 MV/cm the two curves merge and a steady exponential rate of increase in current density is observed for both cases.

In the absence of the blocking oxide layers at either end of the superlattice, carrier injection at the contacts produces a space charge limited current regime^25^. With broadband excitation higher carrier densities can be achieved due to excitons being generated and the current density measures higher^26^. Several mechanisms govern the current density in this initial regime. Due to the small size of the NCs strong Coulomb repulsion limits the current flow below a certain threshold voltage^27,28^. Alongside this mechanism is the discretization of the energy levels within the NCs due to quantum confinement. This, coupled with the fact that there is a small but non-zero variation of size within the ensemble of NCs makes the probability of tunneling between resonant states very low^29^. Therefore, electron–phonon coupling also governs the current density in this regime.

Past 0.1 MV/cm the limit for space charge limited current is reached. However, further increase in current density is observed due to the contribution of a mechanism, whereupon charge transport across the inter-layer-oxide barriers is assisted by surface defects acting as mid-band-gap traps. Unlike conduction band tunneling between two adjacent NCs tunneling from a mid-band gap defect requires an electron to overcome a lower energy barrier and thus the faster charge transport allows current density to be increased.

**Figure 2:**
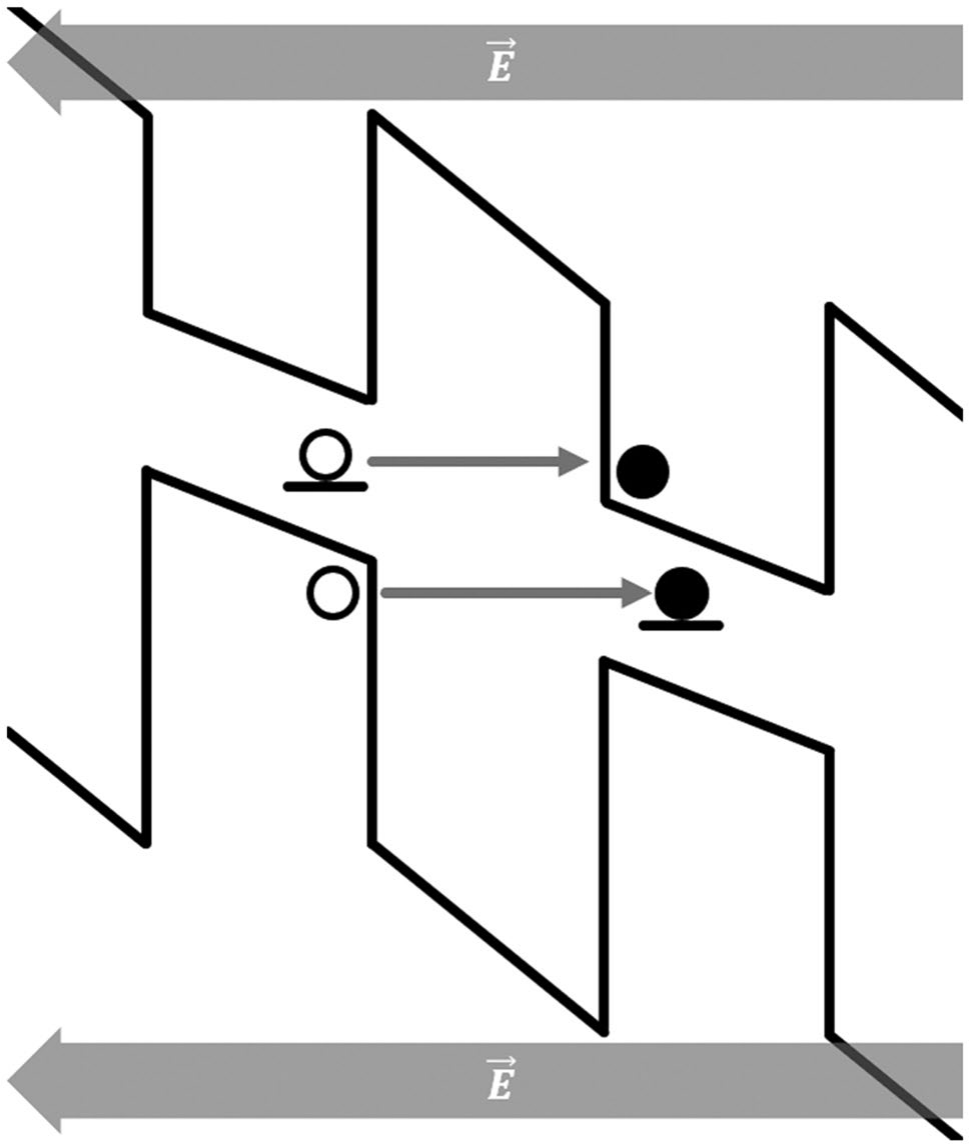
Diagram showing the biased band structure within a section of the superlattice with transitions marked.

In Fig. 2 the tunnelling of a valence band electron to a charged defect site and the transition of an electron from a defect to the conduction band of an adjacent NC are schematically represented. In the case of the measurement under excitation, a higher current density is observed due to the splitting of excitons generating free charge carriers. These carriers migrate across the superlattice adding to the overall current. However, at higher fields this contribution becomes relatively insignificant since the tunneling rate across the inter-layer-oxide barrier is so high that the overall current density is determined by the injection rate alone.

**Figure 3:**
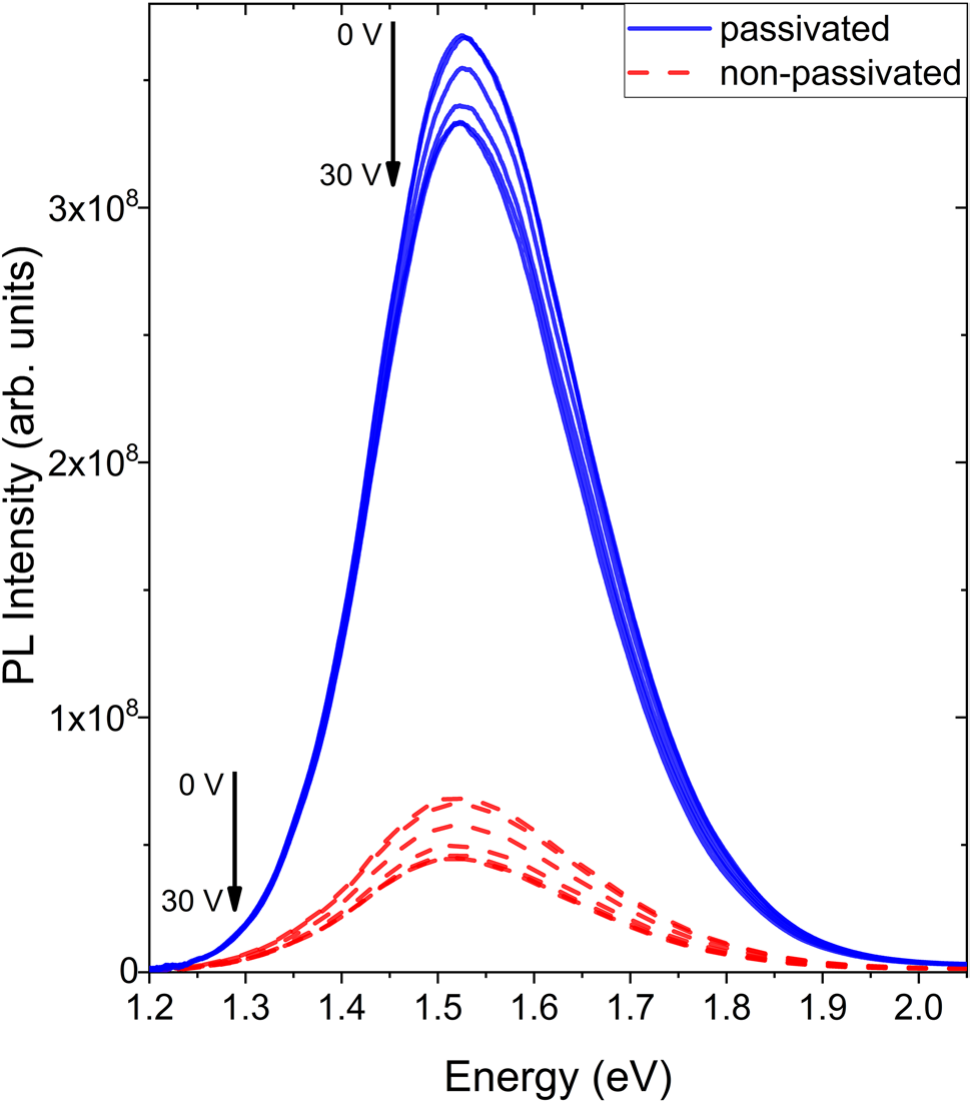
PL spectra as a function of applied voltages (0–30 V, in steps of 5 V) of a passivated and a non-passivated sample with barrier oxide thicknesses of 2 nm measured at RT.

**Figure 4:**
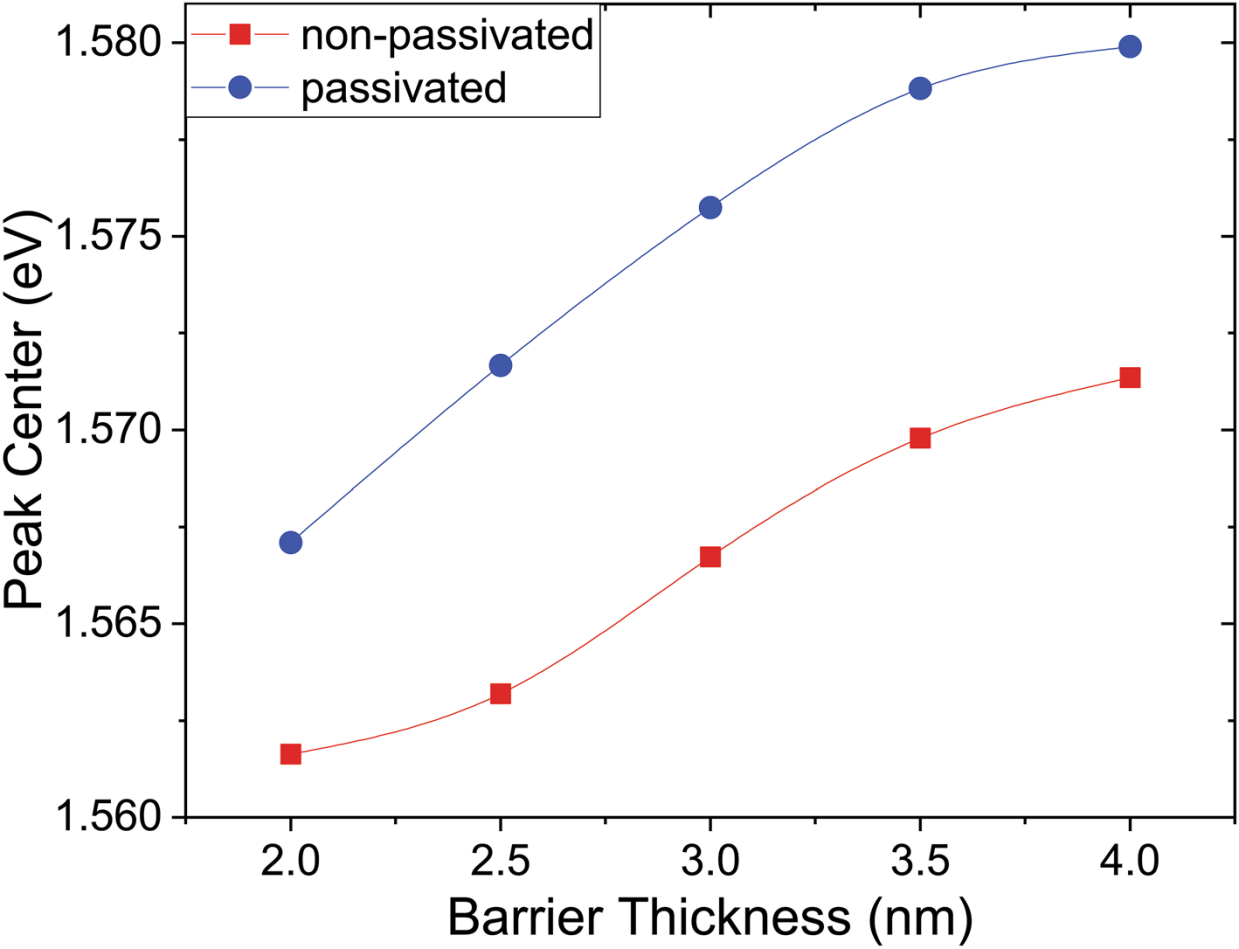
Change in PL peak positions of SiNC samples with varied barrier thicknesses measured at 80 K. The curves connecting the data points are just visual guides.

In Fig. 3 PL spectra of two sets of samples are plotted as a function of the applied voltage comparing the passivated and un-passivated state behavior at RT. A clear decrease of the PL intensity is seen with increasing applied voltage in both cases. The peaks centered around 1.56 eV (795 nm) are characteristic of the quantum confined PL spectra of 3.5 nm NCs^30^. Comparing the PL intensity of the peaks, it is seen that the H_2_ passivation step results in NCs with a luminescence intensity several times larger than that of the non-passivated ones. This enhancement of the PL intensity is due to the reduction of the surface defects at the Si/SiO_2_ interface^31^. These defects act as electron acceptors. Non-radiative recombination in these deep level traps reduce the internal quantum yield. Through H_2_ treatment the NC surfaces at the interfaces are passivated reducing mainly P_b_ defects^32^. Since the density of the interfacial surface defects depends on the ratio of surface atoms to total atoms in a NC the enhancement in luminescence intensity is disproportionately larger for smaller NCs. There might be a shift of the emission peak to higher energies if a small size distribution is still present within the ensemble of NCs. As seen in Fig. 4 there is also a shift towards higher energies as the barrier thickness (i.e., inter layer spacing) is increased pertaining to the weaker coupling of the NC layers^19^.

**Figure 5:**
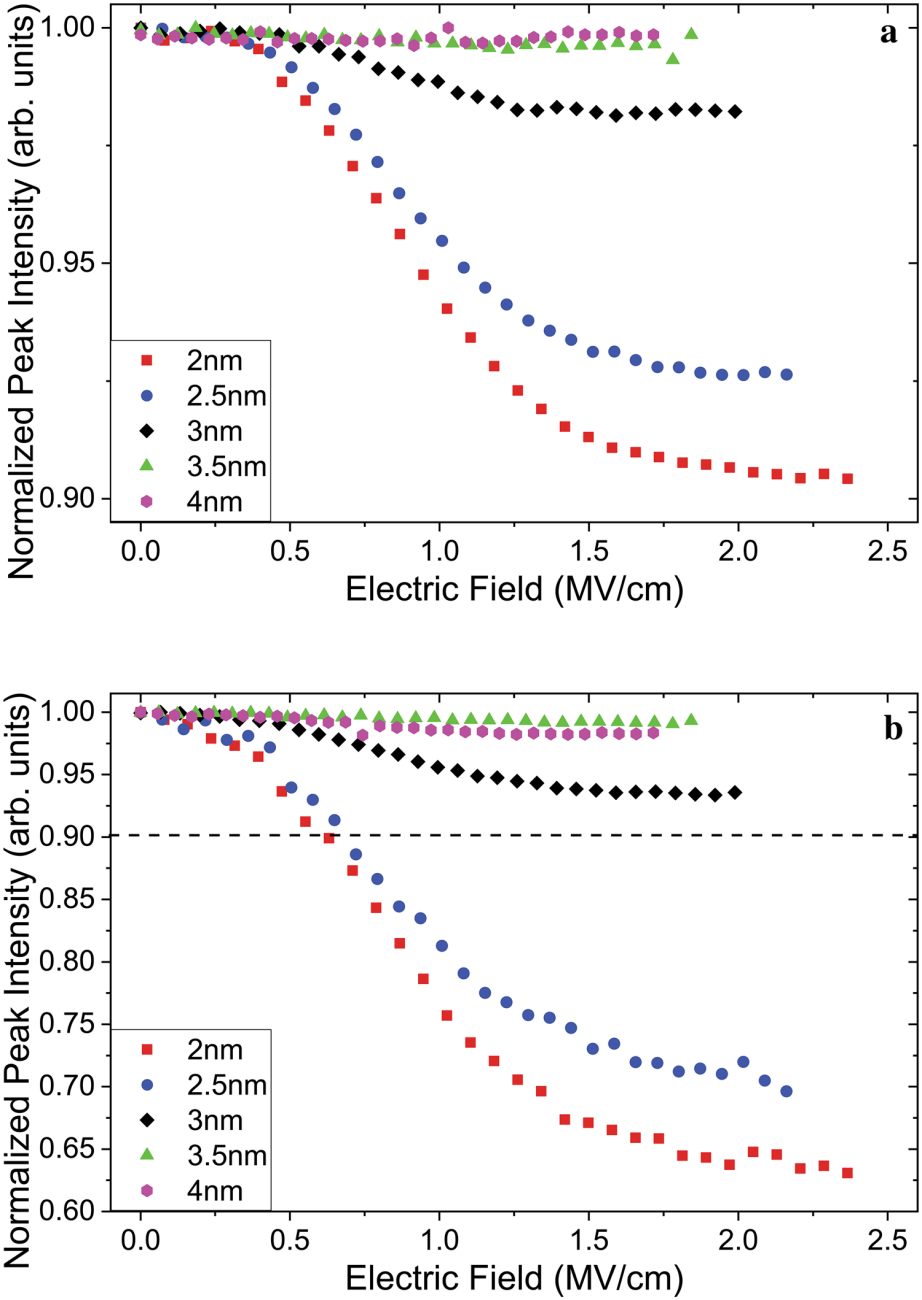
Integrated PL intensities plotted against the electric field for samples with varied inter-layer-oxide barrier thicknesses of the passivated (**a**) and non-passivated (**b**) sample sets, measured at RT. The dashed line in (**b**) marks the lower limit of the vertical axis in (**a**).

**Figure 6:**
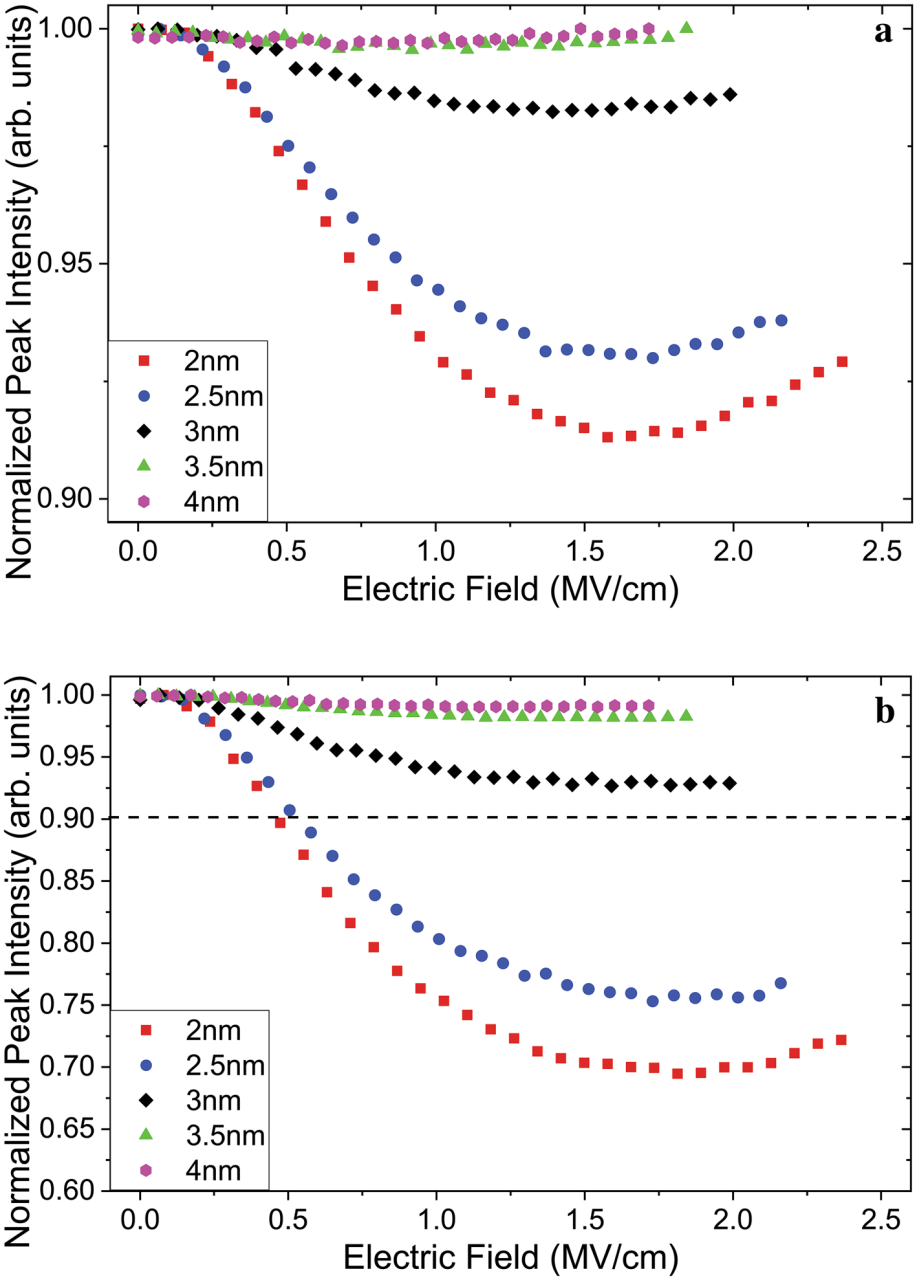
PL intensity–electric field plots as a function of the barrier thickness for the passivated (**a**) and non-passivated (**b**) sample sets, measured at 80 K. The dashed line in (**b**) marks the lower limit of the vertical axis in (**a**).

Similar structures have been shown to exhibit a red shift of the emission under the effect of externally applied electric fields which was explained as a result of the quantum confined Stark effect^33^. This effect becomes significant at higher field strengths but, we did not observe such a trend in the past on our samples for field magnitudes in the range used for the present study^34^. However, what is observed is a significant reduction in the peak intensities as the voltage increases. In Figs. 5 and 6 the changes in PL intensities in relation to increasing field strength are shown as the evolution of the integrated area under the emission peaks.

In both Fig. 5a and b a distinct trend of decreasing integrated PL intensity is seen as the field magnitude is increased. This trend is limited to samples with inter-layer-oxide barrier thicknesses less than 3 nm and is much more pronounced for those with thinner barriers. At field magnitudes surpassing 1.5 MV/cm the curves flatten but the decrease continues, especially for the samples with 2 nm and 2.5 nm barriers in (b). The passivated samples in (a) flatten out the most, where the one with 3 nm barriers shows almost no decrease past 1.75 MV/cm.

While the trends in (a) and (b) are similar, the PL intensity drops to a minimum of 63% for the non-passivated sample with a barrier oxide layer thickness of 2 nm, whereas this value is only 90% for its passivated counterpart. Another significant difference between the two sets of plots is the late onset of the intensity reduction effect on the passivated samples. While the intensity drop starts almost immediately (0.06 MV/cm) for the non-passivated samples, for those that have undergone the H_2_ treatment, no significant reduction is seen until the field strength reaches 0.35 MV/cm.

In Fig. 6 similar trends of decreasing PL intensity at 80 K are seen up to 1.5 MV/cm. Samples with thinner inter-layer-oxide barriers and samples without H_2_ passivation exhibit the highest decrease in luminescence intensity. However, unlike the measurements done at RT these decreasing emission trends in both Fig. 6a and b come to a halt and reverse direction. This effect is seen most clearly for the passivated sample with 2 nm inter-layer-oxide barriers, where the PL intensity at 2.25 MV/cm matches that of 1.0 MV/cm. A comparison between Figs. 5a and 6a, also shows that the onset of the reduction effect is shifted from 0.35 to 0.20 MV/cm as the samples are cooled down to 80 K.

Considering that the dependency of the PL intensity on the applied electric field is found to be conditional on the thickness of the inter-layer-oxide barriers being less than 3 nm, one can reason that charge transfer between the NC layers is regulating this behavior. There is an inverse relation between the thickness of the oxide and the tunneling current^35^. Another supporting argument for this case is provided by the fact that this luminescence reduction effect is enhanced when the surface defects are not passivated by H_2_ treatment. As the current density measurements show, surface defects play a significant role in charge transfer across the NC layers in SiNC superlattices through the trap assisted charge transfer mechanism mentioned earlier. The contribution of this mechanism was also reported in studies concerning charge transport in Si/SiO_2_ superlattices^14^. While this is not the only significant mechanism, the effect of the surface defects on the Si/SiO_2_ interfaces in particular, can be verified in a controlled manner allowing for the confirmation that the luminescent intensity is reduced further if charge transport through the superlattice is enhanced.

This is significant since the reduction in the luminescence can be directly linked to exciton splitting. Without the externally applied field the PL intensity is defined by the absorption cross section and the internal quantum yield of the NCs, which in the ideal case, would result in a one-to-one ratio of generated excitons to emitted photons^36^. With the applied field, a fraction of the excitons can be split and migrate across the superlattice effectively charging NCs. A charged NC can also absorb incident photons, generating hot electrons but since Auger recombination lifetimes are several orders of magnitude shorter than that of PL these NCs would not contribute to PL emission^37,38^.

This is demonstrated by a first order approximation of a rate equation model where the PL and Auger recombination rates are defined in terms of their respective lifetimes (${\tau }_{PL}$ and ${\tau }_{A}$) the density of photo-generated excitons (${N}^{1}$) conduction band carrier density (${D}^{c}$) and the exciton generation rate ($G$):

1 $${I}_{PL}= \int \frac{{N}^{1}}{{\tau }_{PL}} \eta \,dt$$

where

2 $$\frac{d{N}^{1}}{dt}=G-\frac{{N}^{1}}{{\tau }_{PL}}-\frac{{N}^{1}{D}^{c}}{{\tau }_{A}}$$

Note that only the single exciton generation case is considered and the internal quantum yield ($\eta$) is set to one for simplicity. Using experimentally determined literature values for ${\tau }_{PL}$ and ${\tau }_{A}$ at room temperature and solving Eqs. (1) and (2) for an excitation power density of 0.65 mW/cm^2^ with complete absorption yields PL intensity as a function of the conduction band carrier density, ${D}^{c}$^19,39^.

**Figure 7:**
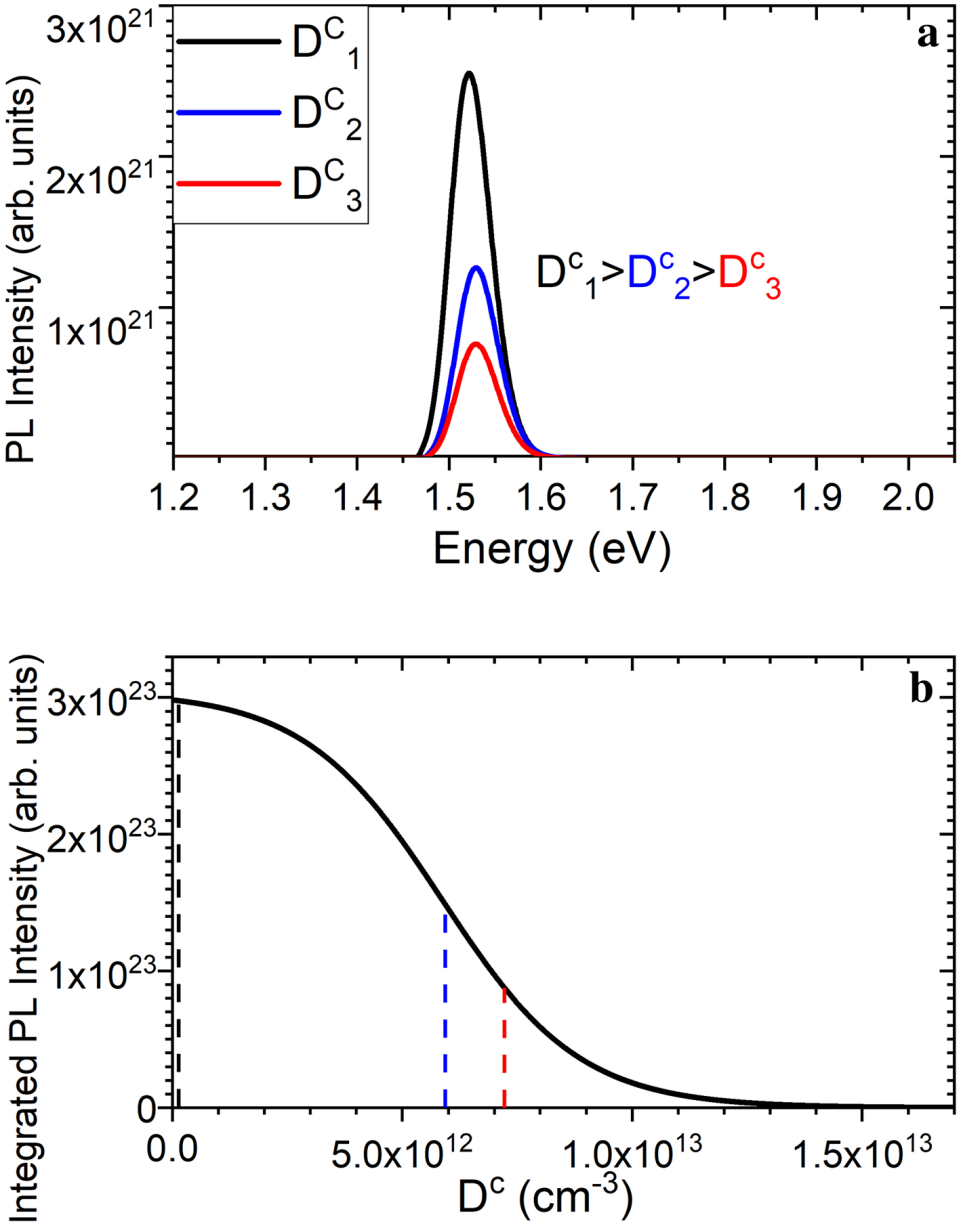
PL spectra calculated for three arbitrary values of D^c^ (**a**) and integrated PL intensity as a function of D^c^ (**b**) (The dashed lines in (**b**) correspond to the spectra in (**a**)).

In Fig. 7a simulated PL spectra for an ensemble of 3.5 nm NCs with a standard deviation of 0.2 nm are plotted using different values for ${D}^{c}$. The emission peak centers were derived from experimental values obtained in previous studies on similar NCs linking the bandgap at 0 K to NC size and the temperature dependent bad gap broadening phenomenon^30,40^.

This approach shows that an increase in the density of conduction band electrons would result in a reduction in PL. Without charging the Auger recombination rate would be significantly lower than that of PL since it is a three-particle interaction and the density of conduction band electrons in intrinsic Si is several orders of magnitude lower than the values in Fig. 7b (7.4 × 10^9^ cm^−3^). In the case of charged NCs the presence of high energy electrons in the conduction band increases the Auger recombination rate and the PL signal is reduced by Auger quenching. Auger recombination would also reduce the conduction band carrier density while exciton splitting charges NCs. The effect seen in the steady state PL measurement is the equilibrium reached between these two mechanisms. In this case the reduction in PL intensity can be ascribed to the fraction charged NCs not contributing to PL at any given time.

There are several factors affecting the onset of exciton splitting. In the case of a sample that has undergone surface passivation only a small amount of surface defects take part in charge transport. The current is mainly regulated by the rate of inter-NC polaronic direct tunneling, therefore a sufficient field strength across the oxide has to be reached for exciton splitting. Without the H_2_ passivation process, the onset is near immediate and the signal reduction effect itself is almost quadrupled. This is ascribed to the trap assisted charge transfer mechanism allowing for much faster charge transport rates through the oxide than direct tunneling alone would. The early onset of the luminescence reduction effect (i.e., exciton splitting) is due to the enhanced carrier transport achieved through the utilization of the surface defects.

**Figure 8:**
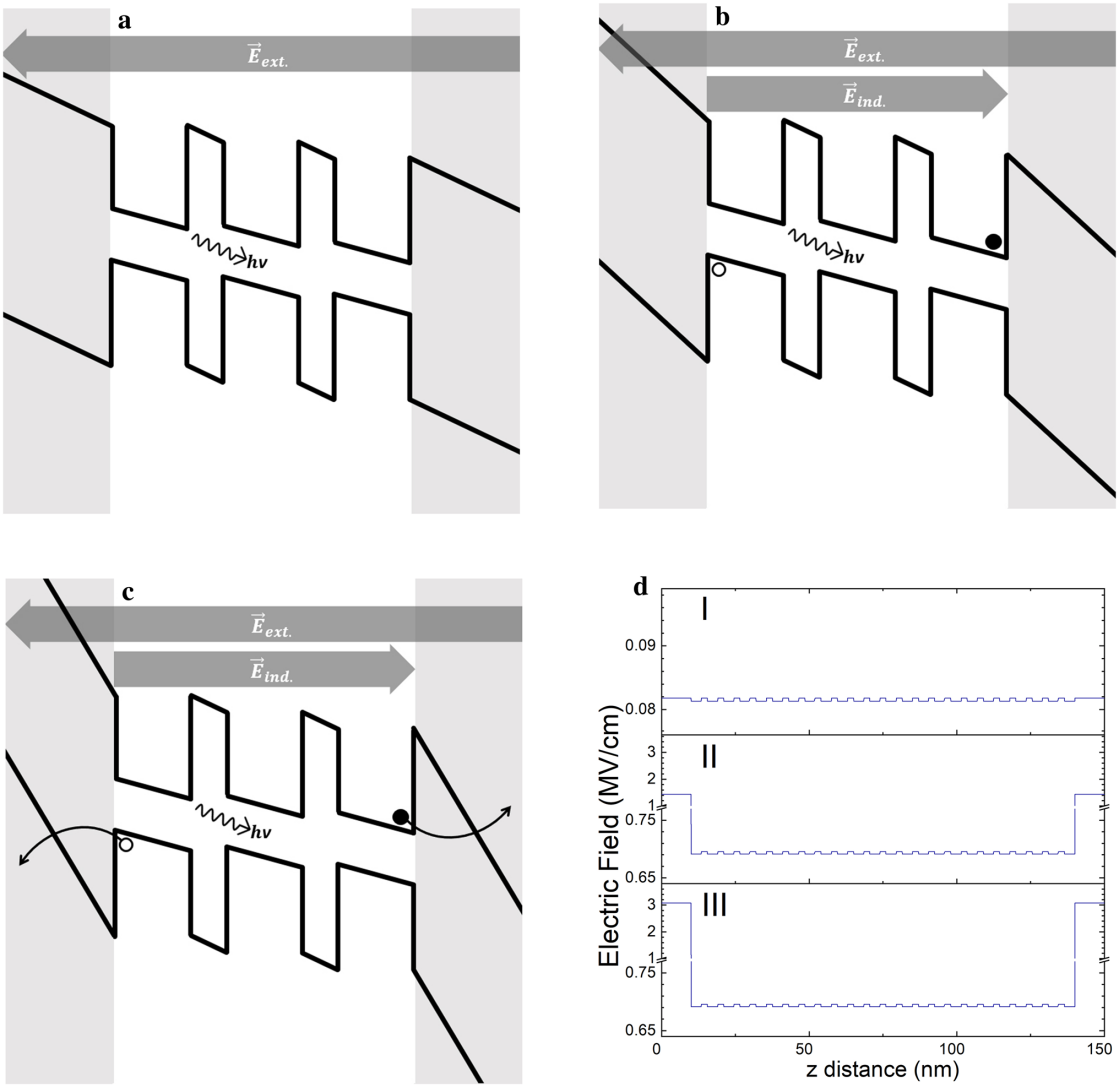
Band structure diagrams across a sample with 2 nm inter-layer-oxide barriers at average external field magnitudes of 0.1 MV/cm (**a**), 1.0 MV/cm (**b**) and 2.0 MV/cm (**c**), with corresponding plots of modelled effective local fields (**d**)-**I**, (**d**)-**II** and (**d**)-**III** plotted as a function of the distance “z” from substrate surface.

However, the carrier transfer enhancement provided by the presence of the defects is temperature dependent. Tunneling of an electron from the conduction band of a NC to a surface defect is accompanied by the absorption of phonons^41^. At 80 K, with the phonons frozen-in, charge transfer rate through this mechanism is severely reduced, explaining the delayed onset of the reduction in PL intensity seen in Fig. 6b. The luminescence reduction, also does not reach the same level when measured at 80 K. Furthermore, the luminescence intensity reaches a minimum around 1.75 MV/cm and recovers. To explain this behavior, the development of the field across the entirety of the sample and within the superlattice needs to be examined in detail, using PL measurements in tandem with a model of the local field magnitudes. In Fig. 8 effective local field and potential values obtained using the experimental parameters with a finite element analysis model are shown. In these the NC layers are considered to have the properties of a bulk medium with an average effective permittivity value equal to the weighted average (by volume) of that of its constituents.

As it is in the case of samples without blocking oxide layers at either end of the superlattice, once the field has reached a threshold value an exciton splitting is achieved. The factors effecting this magnitude of this threshold value is explained above. At fields lower than the exciton splitting threshold, the band structure across the sample is biased as depicted in Fig. 8a. The voltage drops uniformly across the entirety of the sample, with the highest field magnitudes being reached in the oxide at the Si/SiO_2_ interfaces There is no charge accumulation in this regime, no charged NCs and no change in PL intensity.

Past the threshold field value, excitons are split and carriers migrate across the superlattice. These carriers charge NCs, leading to a reduction in PL intensity. Due to the presence of the blocking oxide barriers, charge accumulates at either end of the superlattice. This charge accumulation generates an induced field counteracting the externally applied field. Generation of an induced electric field leads to a reduction on the magnitude of the effective average field “felt” by the NCs in the superlattice. However, this reduction is not unlimited, since it reduces the effect of its own driving force. The value can drop until the average field in the superlattice excluding the blocking oxide layers reach the exciton splitting threshold value, at which point a steady state balance is reached. Since the external field is a controlled experimental parameter, the reduction is compensated for, by the increase of the field across the blocking oxide barriers (cf. Fig. 8d-II. The voltage drop is not uniform and the highest field values are inside the oxide immediately at the interface between the charged NCs and the blocking oxide barriers. In the steady state PL measurements, this correlates to the PL decrease regime that can be seen up to 1.5 MV/cm in Fig. 5a.

At higher fields, this uneven distribution of field strength results in local effective field values in the blocking oxide barriers getting much higher than that of the average field across the entire sample. In Fig. 8d-III it is seen that the field in the blocking oxide reaches 3.24 MV/cm while the average field is only 2.0 MV/cm. At these high field values the accumulated charge flows into the contacts through field emission of electrons across the 10 nm blocking oxide. In the steady state, this results in a smaller number of charged NCs leading to a flattening of the reduction in Fig. 5a and the recovery of the PL intensity in Fig. 6a.

Exciton splitting energies can be calculated using the threshold field magnitudes extracted from Figs. 5 and 6 by extrapolating the decreasing trend with a linear curve fit and taking the intersection at unity as the threshold. The threshold field value (**F**_s_) in this case, is the field value necessary for exciton splitting and does not get reduced by the superimposed internal field.

3 $${E}_{split}= {{\varvec{F}}}_{s}\, {d}_{stack }\,{q}_{e}$$

where d_stack_ is the total thickness of all the NC layers and barriers in between and q_e_ is the elementary charge. Here, unlike the exciton binding energy as an intrinsic property of the bulk material, exciton splitting energy is defined as the energy required to split an exciton and transfer the charge across the superlattice, which includes the exciton binding energy but also the work done to move the charges across the superlattice.

**Figure 9:**
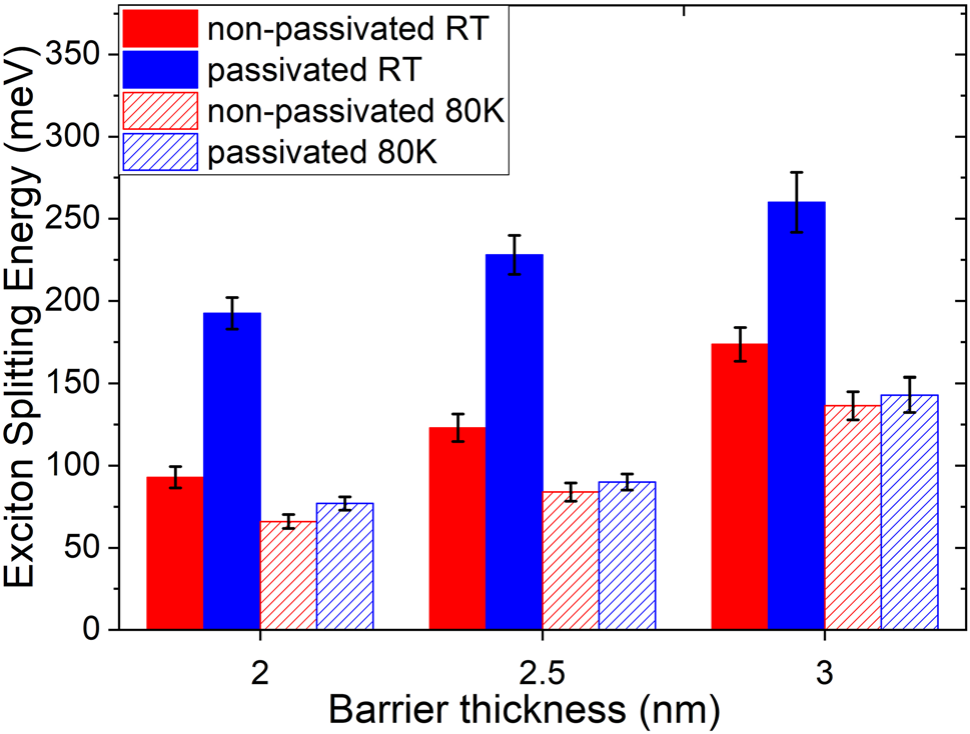
Calculated exciton splitting energies for NCs as a function of inter-layer-oxide barrier thicknesses.

These values in Fig. 9 show that under all experimental conditions there exists a correlation between barrier thickness and increasing exciton splitting energy. This phenomenon is explained considering the polarization of the dielectric as an electron is transferred between two NCs. With shorter inter layer distances (i.e., barrier thickness) a thinner section of dielectric needs to be polarized by a migrating electron. This reduction of the work needed results in lower exciton splitting energies for samples with thinner barrier oxides. On the other end of this scale, for samples with barrier oxides thicker than 3 nm, the exciton splitting energies are so high that no PL reduction effect is observed. It is also seen that the exciton splitting energies are invariably lower at 80 K for all samples. This can be explained by the dependence of the relative permittivity of the medium on temperature. The exciton radius scales linearly with the permittivity of the medium therefore higher permittivity at lower temperatures reduces the exciton splitting energy^42^.

Ultimately, through the analyses of PL measurements, it was shown that the application of an electric field across a SiNC superlattice under excitation causes exciton splitting and the charging of NCs with these free charge carries. Charged NCs accumulate at either end of the superlattice and do not contribute to PL. In the steady state measurements, this is measured as the reduction of luminescence intensity. At higher field magnitudes field emission transfers the accumulated charge to the contacts. With the introduction of contacts between the blocking oxide layers and the superlattice itself, this platform can potentially be used as a photo-detector where the absorption band edge can be tailored by exploiting the quantum confinement effect. The exciton splitting and accompanying charge accumulation requires a tunneling current across the barrier oxide which was found to be enhanced by a trap-assisted transfer mechanism. This enhancement allows for carrier splitting at lower fields in NCs with higher surface defect densities. The exciton splitting threshold in NCs is also influenced by temperature where the trap assisted charge transfer mechanism is hindered at low temperatures due to the freezing in of phonons. Lowest exciton splitting thresholds can be achieved at room temperature without surface passivation. However, it should be noted that for photovoltaics applications surface passivation is still necessary. The current density enhancement provided by the increased surface defect density is not within the range of the enhancement in quantum yield provided by the lowered surface defect density the passivation process achieves^43^. Considering the case of a tandem solar cell in series, the potential across the superlattice would have be limited to below 1.12V^44,45^. To achieve the necessary 0.35 MV/cm field strength the thickness of the NC top cell would have to be below 32 nm, allowing for a maximum of five bilayers. With these conditions met, higher yield could be achieved without limiting the short-circuit current of the tandem device. These values apply only to the specific case of the structures in the present study. Using the same experimental method, these values can be determined for any Si/SiO_2_ superlattice with different barrier thicknesses and NC size.

## Data Availability

The data that support the findings of this study are available from the corresponding author upon reasonable request.
